# Mannose-modified hemocyanin enhances pathogen endocytosis by crustacean hemocytes

**DOI:** 10.1016/j.jbc.2025.110304

**Published:** 2025-05-28

**Authors:** Jiaxi Li, Jude Juventus Aweya, Mingming Zhao, Yongzhen Zhao, Zhongyang Lin, Xiuli Chen, Zhihong Zheng, Pengfei Li, Defu Yao, Yueling Zhang

**Affiliations:** 1 Guangdong Provincial Key Laboratory of Marine Biotechnology, Institute of Marine Sciences, Shantou University, Shantou, China; 2 Guangxi Key Laboratory of Aquatic Biotechnology and Modern Ecological Aquaculture, Guangxi Engineering Research Center for Fishery Major Diseases Control and Efficient Healthy Breeding Industrial Technology (GERCFT), Guangxi Academy of Sciences, Nanning, China; 3 Department of Food and Human Nutritional Sciences, University of Manitoba, Winnipeg, Manitoba, Canada; 4 The Canadian Centre for Agri-Food Research in Health and Medicine, St Boniface Hospital Albrechtsen Research Centre, Winnipeg, Manitoba, Canada; 5 Guangxi Academy of Fishery Sciences, Guangxi Key Laboratory of Aquatic Genetic Breeding and Healthy Aquaculture, Nanning, China

**Keywords:** cellular immune response, endocytosis, invertebrate, membrane trafficking, post-translational modification (PTM), protein translocation, receptor endocytosis, crustaceans, hemocyanin

## Abstract

In crustaceans, hemolymph plasma contains more than 90% hemocyanin, whereas hemocytes have minimal levels, suggesting a regulated uptake mechanism. Here, we demonstrate that in *Penaeus vannamei*, hemocytes internalize plasma hemocyanin under normal conditions *via* phagocytosis, clathrin-mediated endocytosis, and micropinocytosis. This uptake is significantly enhanced during bacterial (*Vibrio parahaemolyticus*, *Vibrio alginolyticus*, *Staphylococcus aureus*, *Streptococcus iniae*) and viral (White spot syndrome virus) infections or upon stimulation with pathogen-associated molecular patterns. While post-translational modifications (PTMs) such as dephosphorylation, deacetylation, and mannosylation enhance hemocyanin’s pathogen-binding affinity, only mannosylation promotes mannose receptor-mediated endocytosis for intracellular clearance, whereas dephosphorylation and deacetylation facilitate extracellular pathogen elimination. These findings reveal that hemocyanin functions beyond oxygen transport, acting as an immune effector that undergoes PTMs to enhance intracellular pathogen clearance.

The hostile biotic and abiotic factors in the aquatic environment demands that aquatic organisms adopt various survival strategies (1). Moreover, climate change and anthropogenic activities compound the complex aquatic ecosystem, consequently exerting environmental stress on most organisms, especially marine invertebrates such as crustaceans, affecting their innate immune systems and disease susceptibility (2). In the context of escalating environmental challenges such as oceanic warming, acidification, and excessive pollution, the innate immune systems of invertebrates, notably crustaceans, are undergoing modifications as a result of both active and passive adaptations to environmental alterations (3). These evolutionary changes in immune regulatory mechanisms are poised to have a direct impact on their efficacy in defending against pathogenic infections. In general, when crustaceans encounter pathogen invasion, its innate immune system is triggered upon pattern recognition receptors (PRRs) recognizing and binding to pathogen-associated molecular patterns (PAMPs) (4, 5, 6), a cascade of events occur to clear the evading pathogen, including the activation of various signaling pathways and immune response processes, such as coagulation, phagocytosis, apoptosis, and so on, and the expression and release of numerous immune effectors (7, 8).

Many environmental stress conditions can challenge cellular life; hence, most cells have developed complex stress-coping responses. Moreover, the cell membrane, the first point of encounter with many environmental stress factors (*e*.*g*., microbial pathogens), expresses various receptor proteins and their associated molecules that help to respond to these factors through mechanisms such as endocytosis (9, 10, 11). Thus, endocytosis is vital in the cellular uptake of nutrients, clearance of pathogens and apoptotic cells, cell adhesion and migration, signaling, antigen presentation, and *so on* (11). Depending on cellular needs and cell membrane morphology, various forms of endocytosis are involved, including macropinocytosis, phagocytosis, clathrin-mediated endocytosis (CME), caveolae-mediated endocytosis (CvME), and clathrin/caveolin-independent endocytosis (11, 12).

As a cellular process, endocytosis reacts to external stimuli accordingly, including high temperature (13), heavy metals (14), peroxides (15), and nanoparticles (16). Similarly, internal factors, such as immune signaling pathways through opsonin binding to pathogens, C-type lectin, complement, immunoglobulin, antimicrobial peptides, *etc*., as well as an appropriate amount of free radicals (15), certain polysaccharides (17), and pathogens (18) can promote cellular endocytosis, such as phagocytosis.

Phagocytosis is a crucial part of the innate immune response as it helps to engulf and eliminate pathogens, induce signaling pathways, secrete immune factors, take part in antigen presentation, and so on (19, 20). In crustaceans, hemocytes are regarded as professional phagocytes (21) that play various roles in cellular immunity, such as encapsulation, coagulation, melanization, and phagocytosis. Endocytosis is, therefore, a critical part of the innate immunity of crustaceans, although, unlike mammalian phagocytes, there is still a dearth of information on molecular marker proteins in crustacean hemocytes (21, 22). Nonetheless, in the marine gastropod mollusk *Concholepas concholepas*, it has been reported that the respiratory protein hemocyanin can be internalized and catabolized by hemocytes to produce immune and metabolic factors, such as antibacterial peptides and phenoloxidase (23). Molluscan hemocyanin has also been shown to be recognized and internalized by receptors on mammalian immune cells, resulting in the activation of cellular immune responses. The binding of hemocyanin to Toll-like receptor 4 (TLR4) activates the associated signaling pathways to generate pro-inflammatory responses in antigen-presenting cells (24). On the other hand, the binding of hemocyanin to the mannose receptor (MR) and DC-specific ICAM-3-grabbing nonintegrin (DC-SIGN) on dendritic cells results in its uptake into the cells through clathrin-mediated endocytosis (25). Similarly, when *N*-glycosylated hemocyanin binds to MR and macrophage galactose lectin (MGL) on mammalian macrophages, it is uptaken into cells to promote the expressions of tumor necrosis factor α and interleukins 6 and 12 (26). Our preliminary studies in penaeid shrimp revealed that hemocyanin could be internalized by hemocytes, especially during pathogen challenge. However, the mechanism by which hemocyanin is internalized and its immunological and/or pathophysiological importance is unknown.

Here, we determined the mechanism by which *Penaeus vannamei* hemocytes internalize hemocyanin from plasma. We show that shrimp hemocytes could endocytose hemocyanin through phagocytosis, clathrin-mediated endocytosis, and micropinocytosis, especially when challenged with microbial pathogens. Moreover, the mannose receptor, a member of the C-type lectin family, is a crucial mediator in the phagocytosis of hemocyanin, modifying hemocyanin to enhance it binding with microbial pathogens for intracellular clearance, a vital part of shrimp's cellular immune response.

## Results

### Hemocytes and macrophage-like cells internalize shrimp plasma hemocyanin

**Figure 1:**
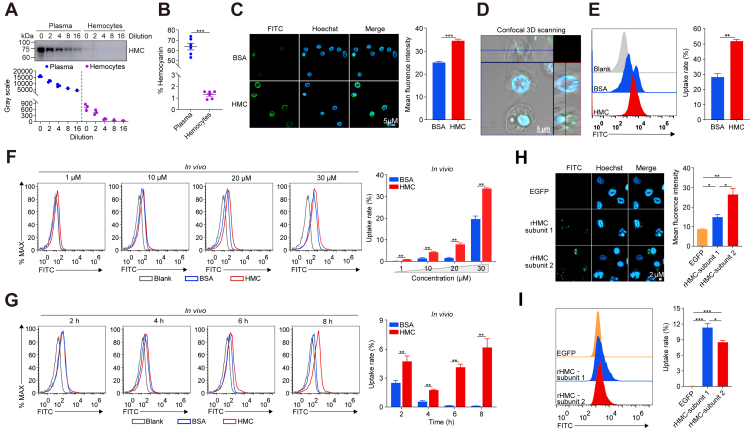
**Hemocytes internalize plasma hemocyanin**. *A*, Western blot analysis of hemocyanin (HMC) levels in diluted plasma and hemocyte lysates (*top*), with relative band intensity quantified using ImageJ (*bottom*). *B*, relative HMC protein expression in plasma and hemocytes determined by spectrophotometry. *C*, confocal microscopy images showing endocytosis of FITC-labeled hemocyanin (FITC-HMC) and FITC-labeled bovine serum albumin (FITC-BSA) by hemocytes. Scale bar = 10 μm. Nuclei stained with Hoechst 33,342 (*blue*). Mean fluorescence intensity was quantified using ImageJ. *D*, Representative 3D reconstruction of internalized FITC-HMC. Scale bar = 5 μm. *E*, relative endocytosis of FITC-HMC and FITC-BSA by hemocytes analyzed *via* Flow cytometry, quantified using FlowJo. *F*, endocytosis of FITC-HMC and FITC-BSA in hemocytes after *in vivo* treatment. *G*, Time-course analysis (2, 4, 6, and 8 h) of FITC-HMC and FITC-BSA uptake following *in vivo* treatment. *H*, confocal images of hemocytes internalizing EGFP, recombinant hemocyanin subunit 1 (rHMC-subunit 1), and subunit 2 (rHMC-subunit 2). Scale bar = 2 μm. Nuclei stained with Hoechst 33,342 (*blue*). Mean fluorescence intensity was quantified using ImageJ. *I*, relative endocytosis of EGFP, rHMC-subunit 1, and rHMC-subunit 2 by hemocytes, analyzed *via* flow cytometry and quantified using FlowJo. Results are presented as mean ± SEM (n = 3). ∗*p* < 0.05, ∗∗*p* < 0.01, ∗∗∗*p* < 0.001 vs. control. Error bars represent S.E. Immunoblots and microscopy images are representative of at least three independent experiments. BSA, bovine serum albumin; EGFP, enhanced *green* fluorescent protein; FITC-BSA, FITC-labeled bovine serum albumin; FITC-HMC, FITC-labeled hemocyanin, HMC, hemocyanin; rHMC-subunit 1, EGFP-labeled recombinant hemocyanin subunit 1; rHMC-subunit 2, EGFP-labeled recombinant hemocyanin subunit 2.

Hemocyanin accounts for over 90% of total plasma proteins in crustacean hemolymph (27, 28), a finding we confirmed in *P*. *vannamei* (Fig. 1, *A* and *B*). To assess whether hemocytes internalize purified endogenous hemocyanin, primary shrimp hemocytes were treated with FITC-labeled endogenous hemocyanin (HMC-FITC) or FITC-labeled BSA (BSA-FITC) as a control and analyzed using a laser scanning confocal microscope (LSCM) and flow cytometry (FACS). HMC-FITC-treated hemocytes exhibited significantly higher fluorescent intensity (*p* < 0.05) compared to BSA-FITC-treated cells (Fig. 1*C*). Three-dimensional LSCM imaging (Fig. 1*D*), and the FACS analysis further confirmed significant internalization of HMC-FITC (*p* < 0.05) compared with BSA-FITC (Fig. 1*E*). Additionally, hemocyanin uptake was consistently higher than BSA across all tested concentrations (*p* < 0.01) (Fig. 1*F*). Over time, hemocytes continued engulfing hemocyanin, whereas BSA internalization gradually declined (*p* < 0.01) (Fig. 1*G*). Similarly, macrophage-like cells, including *Drosophila melanogaster* S2 cells and mouse RAW264.7 cells, internalized significantly more HMC-FITC than BSA-FITC (Fig. S1, *A*–*C*). These results indicate that shrimp hemocytes and macrophage-like cells can internalize purified endogenous hemocyanin under normal physiological conditions.

To rule out potential false-positive results associated with FITC labeling, we treated hemocytes with two EGFP-tagged recombinant hemocyanin subunits (rHMC-subunit one and rHMC-subunit 2) or EGFP alone as a control. LSCM and FACS analyses revealed significantly higher (*p* < 0.05) uptake of both rHMC-subunit one and rHMC-subunit two compared to EGFP (Fig. 1, *H* and *I*). Although the mean fluorescence intensity of rHMC-subunit one was significantly lower (*p* < 0.05) than rHMC-subunit 2 (Fig. 1*H*), its overall uptake by hemocytes was significantly higher (*p* < 0.05) (Fig. 1*I*). Similar trends were observed in *D*. *melanogaster* S2 and RAW264.7 cells, where internalization rates and fluorescence intensities for both recombinant subunits were significantly higher (*p* < 0.05) than EGFP (Fig. S1, *E*–*H*), albeit with slight differences between rHMC-subunit one and rHMC-subunit 2. These results suggest that hemocyanin internalization is influenced by its subunit composition.

### Multiple endocytic pathways mediate hemocyanin internalization

**Figure 2:**
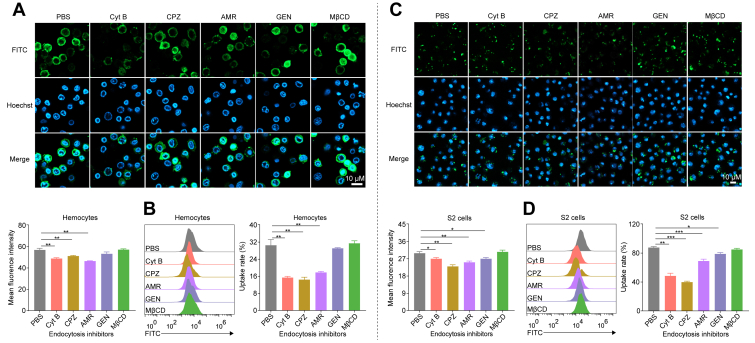
**Hemocyanin uptake occurs *via* multiple endocytic pathways.***A*, confocal microscopy images showing endocytosis of FITC-HMC by (*A*) shrimp hemocytes following treatment with endocytosis inhibitors: cytochalasin B (Cyt B, actin-dependent phagocytosis inhibitor), chlorpromazine (CPZ, clathrin-mediated endocytosis inhibitor), amiloride (AMR, macropinocytossis inhibitor), genistein (GEN, caveolae-mediated endocytosis inhibitor), and methyl-β-cyclodextrin (MβCD, lipid raft inhibitor). PBS treatment served as the control. Scale bar = 10 μm. Nuclei were stained with Hoechst 33,342 (*blue*). Mean fluorescence intensity was quantified using ImageJ. *B*, relative endocytosis of FITC-HMC in shrimp hemocytes treated with inhibitors (Cyt B, CPZ, AMR, GEN, and MβCD), or PBS as control, analyzed by flow cytometry and quantified using FlowJo. *C*, confocal microscopy images showing endocytosis of FITC-HMC by *Drosophila* S2 cells following treatment with endocytosis inhibitors (Cyt B, CPZ, AMR, GEN, and MβCD). PBS treatment served as the control. Scale bar = 10 μm. Nuclei were stained with Hoechst 33,342 (*blue*). Mean fluorescence intensity was quantified using ImageJ. *D*, relative endocytosis of FITC-HMC in *Drosophila* S2 cells treated with inhibitors (Cyt B, CPZ, AMR, GEN, and MβCD), or PBS as control, analyzed by flow cytometry and quantified using FlowJo. Results presented as mean ± SEM (n = 3). ∗*p* < 0.05, ∗∗*p* < 0.01, ∗∗∗*p* < 0.001 vs. control. Error bars represent S.E. Microscopy images are representative of at least three independent experiments. FITC-HMC, FITC-labeled hemocyanin; HMC, hemocyanin.

To identify the endocytic pathways involved in hemocyanin uptake, hemocytes were pretreated with five classical endocytosis inhibitors: Cyt B (phagocytosis inhibitor), CPZ (clathrin-mediated endocytosis inhibitor), AMR (micropinocytosis inhibitor), GEN (caveolae-mediated endocytosis inhibitor), and MβCD (lipid-raft mediated endocytosis inhibitor) before incubation with HMC-FITC or BSA-FITC. None of these inhibitors significantly affected hemocyte viability (Fig. S2*A*). However, Cyt B, CPZ, and AMR significantly reduced (*p* < 0.05) HMC-FITC internalization compared to the control and other inhibitors (Fig. 2, *A* and *B*), indicating that shrimp hemocytes primarily internalize hemocyanin *via* phagocytosis, clathrin-mediated endocytosis, and micropinocytosis.

Further confirmation using additional pathway-specific inhibitors, *i*.*e*., latrunculin A (Lat A, phagocytosis inhibitor), Pitstops 2 (clathrin inhibitor), and ethylisopropylamiloride (EIPA, micropinocytosis inhibitor), also significantly reduced hemocyanin internalization (Fig. S1, *E*–*H*). Similarly, *D*. *melanogaster* S2 and RAW264.7 cells pretreated with these inhibitors exhibited significantly attenuated (*p* < 0.05) uptake of HMC-FITC or BSA-FITC compared with untreated controls (Fig. 2*C* and S2*D*). Notably, RAW264.7 cells showed a marked reduction in HMC-FITC internalization across all tested inhibitors (Fig. S2, *B* and *C*), reinforcing the involvement of multiple endocytic pathways in hemocyanin uptake by macrophage-like cells.

### Hemocyanin enhances the endocytosis of microbial pathogens

**Figure 3:**
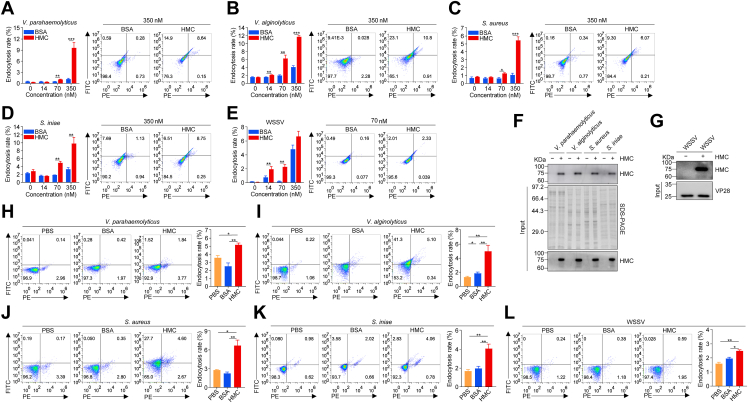
**Hemocyanin enhances the endocytosis of microbial pathogens**. *A-E*, relative shrimp hemocytes endocytosis of DiL-labeled (*A*) *Vibrio parahaemolyticus*, (*B*) *V*. *alginolyticus*, (*C*) *Staphylococcus aureus*, (*D*) *Streptococcus iniae*), and (*E*) *White* spot syndrome virus (WSSV) pre-incubated with varying concentration (0, 14, 70, and 350 nM) of FITC-HMC or FITC-BSA, analyzed by flow cytometry and quantified using FlowJo. *F*, Western blot and SDS-PAGE analysis of hemocyanin binding with *V*. *parahaemolyticus*, *V*. *alginolyticus*, *S*. *aureus*, and *S*. *iniae*. *G*, Western blot and SDS-PAGE analysis of hemocyanin binding with WSSV. *H-L*, Relative hemocytes endocytosis of DiL-labeled (*H*) *V*. *parahaemolyticus*, (*I*) *V*. *alginolyticus*, (*J*) *S*. *aureus*, (*K*) *S*. *iniae*, and (*L*) WSSV following injection of shrimp with 350 nM FITC-HMC or FITC-BSA pretreatment (for bacteria) and 70 nM FITC-HMC or FITC-BSA (for WSSV), analyzed by flow cytometry. Results presented as mean ± SEM (n = 3). ∗*p* < 0.05, ∗∗*p* < 0.01, ∗∗∗*p* < 0.001 vs. control. Error bars represent S.E. Immunoblot images are representative of at least three independent experiments. BSA, bovine serum albumin; FITC-BSA, FITC-labeled bovine serum albumin; FITC-HMC, FITC-labeled hemocyanin; HMC, hemocyanin.

Since endocytosis plays a key role in pathogen clearance (29), we investigated whether hemocyanin uptake by hemocytes (both *in vivo* and *in vitro*) contributes to shrimp’s antimicrobial immune response. Hemocytes were pre-incubated with varying doses of HMC-FITC, BSA-FITC, or PBS before exposure (*in vivo* and *in vitro*) to bacterial (*Vibrio parahaemolyticus*, *Vibrio alginolyticus*, *Staphylococcus aureus*, and *Streptococcus iniae*) and viral (WSSV) pathogens. HMC-FITC pre-incubation led to a significant, dose-dependent increase in pathogen internalization compared to BSA-FITC, PBS, or BSA controls (Fig. 3, *A*–*E*). Similarly, pre-incubation with pathogen-associated molecular patterns (PAMPs), *i*.*e*., LPS, PGN, and LTA, enhanced hemocyanin (HMC-FITC) uptake by hemocytes both *in vivo* and *in vitro* compared with the controls (Fig. S3, *A*–*B*). These results indicate that pathogen or PAMP stimulation enhances hemocyanin internalization by hemocytes.

To determine whether hemocyanin directly binds to microbial pathogens to facilitate endocytosis, bacterial and viral pathogens were incubated with purified hemocyanin and analyzed *via* SDS-PAGE and Western blot. Strong hemocyanin binding was observed across all tested pathogens, with bacteria displaying varying affinities (Fig. 3, *F* and *G*), suggesting that hemocyanin functions as an opsonin to enhance pathogen uptake by hemocytes. Interestingly, while a low dose of HMC-FITC (70 nM) significantly increased WSSV endocytosis, a higher dose (350 nM) markedly attenuated it (*p* < 0.01) (Fig. 3*L*). *In vivo*, shrimp injected with WSSV pre-incubated with 70 nM HMC-FITC exhibited significantly higher WSSV endocytosis than controls (Fig. 3*L*). Additionally, WSSV viral loads and VP28 envelope protein expression were significantly elevated in hemocytes following both *in vitro* and *in vivo* HMC-FITC treatment compared to controls (Fig. S3, *C*–*E*). These results further confirm the enhanced internalization of hemocyanin by hemocytes in response to WSSV infection.

### Endocytic inhibitors attenuate hemocytes’ internalization of hemocyanin-pretreated microbial pathogens

**Figure 4:**
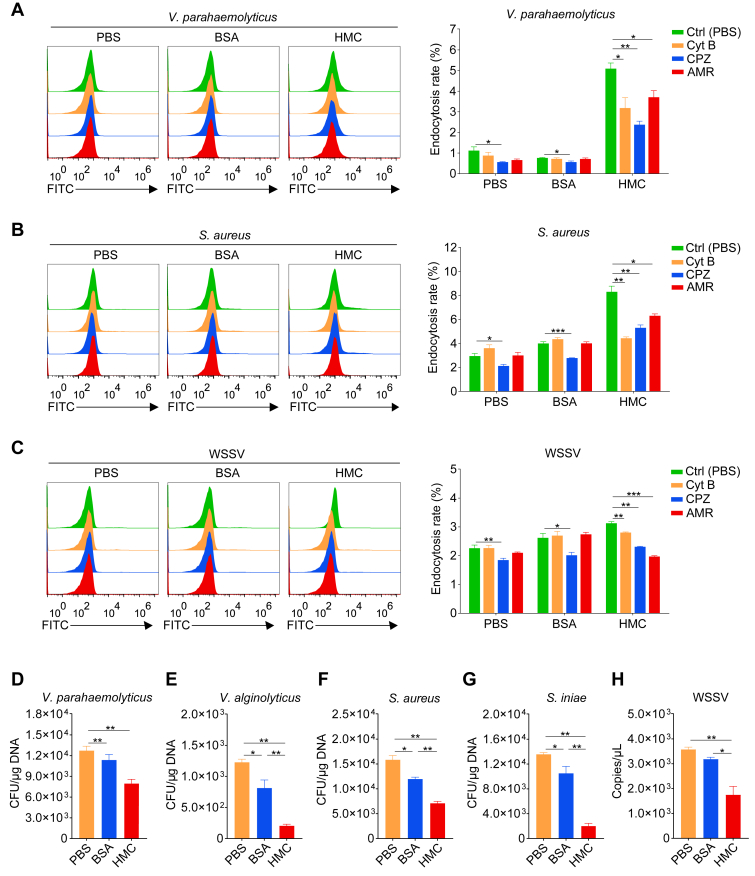
**Hemocyanin enhances microbial clearance by hemocytes**. *A-C*, relative shrimp hemocytes endocytosis of DiL-labeled (*A*) *Vibrio parahaemolyticus*, (*B*) *Staphylococcus aureus*, and (*C*) *white* spot syndrome virus (WSSV) pre-incubated with hemocyanin (HMC) or bovine serum albumin (BSA) treated with endocytosis inhibitors (Cyt B, CPZ, and AMR), and analyzed by flow cytometry. *D-H*, quantification of microbial loads in shrimp hemolymph using real-time PCR following injection with (*D*) *V*. *parahaemolyticus*, (*E*) *V*. *alginolyticus*, (*F*) *S*. *aureus*, (*G*) *Streptococcus iniae*, and (H) WSSV pre-incubated with HMC or BSA. Results are presented as mean ± SEM (n = 3). ∗*p* < 0.05, ∗∗*p* < 0.01, ∗∗∗*p* < 0.001 vs. control. Error bars represent S.E. BSA, Bovine serum albumin; HMC, hemocyanin.

To determine whether hemocyanin-mediated pathogen uptake occurs *via* endocytosis, shrimp were pretreated with endocytosis inhibitors Cyt B, CPZ, and AMR before being challenged with *V*. *parahaemolyticus*, *S*. *aureus*, WSSV pre-incubated with hemocyanin. Control groups received PBS or BSA treatments. Inhibitor-treated hemocytes exhibited significantly reduced internalization of *V*. *parahaemolyticus*, *S*. *aureus*, and WSSV compared to untreated controls, with variations depending on the inhibitor and pathogen type (Fig. 4, *A*–*C*).

In *in vivo* analyses, shrimp hemolymph showed significantly lower relative abundances of *V*. *parahaemolyticus*, *V*. *alginolyticus*, *S*. *aureus*, and *S*. *iniae* in hemocyanin-pretreated samples compared to controls (Fig. 4, *D*–*G*). Interestingly, pre-incubation of WSSV with hemocyanin significantly reduced *in vivo* viral copies in hemocytes compared to control samples (Fig. 4*H*). These results confirm that hemocyanin enhances the endocytic uptake of bacterial and viral pathogens during infections.

### Post-translational modification enhances hemocyanin’s pathogen binding and endocytosis by hemocytes

**Figure 5:**
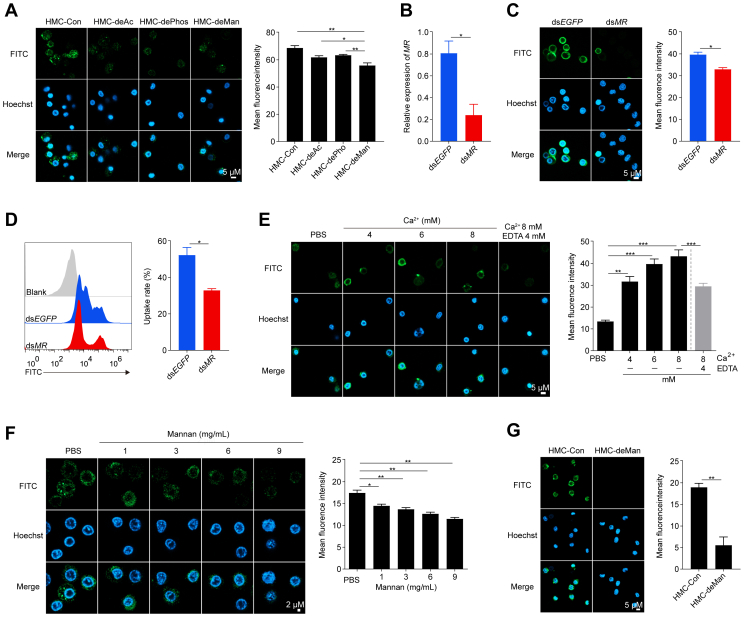
**Mannose receptor mediates hemocyanin endocytosis**. *A*, microscopy images showing hemocytes that have endocytosed FITC-labeled hemocyanin with different post-translational modifications: control hemocyanin (HMC-Con), deacetylated hemocyanin (HMC-deAc), dephosphorylated hemocyanin (HMC-dePhos), and demannosylated hemocyanin (HMC-deMan), as observed *via* laser confocal microscopy. Scale bar = 5 μm. Nuclei are stained with Hoechst 33,342 (*blue*). Mean fluorescence intensity was quantified using ImageJ. *B*, mRNA expression of the mannose receptor (*MR*) in hemocytes following dsRNA-mediated knockdown of *MR*. dsRNA targeting *EGFP* was used as a control. *C*, microscopy images of hemocytes showing FITC-HMC endocytosis after MR knockdown. Scale bar = 5 μm. Nuclei are stained with Hoechst 33,342 (*blue*). Mean fluorescence intensity was quantified using ImageJ. *D*, relative endocytosis of FITC-HMC by hemocytes following MR knockdown, as determined by flow cytometry. Uptake rates were quantified using FlowJo software. *E*, microscopy images of FITC-HMC endocytosis by hemocytes pre-treated with 4, 6, or 8 mM Ca^2+^ or 8 mM Ca^2+^ with 4 mM EDTA. Scale bar = 5 μm. Nuclei are stained with Hoechst 33,342 (*blue*). Mean fluorescence intensity was quantified using ImageJ. *F*, microscopy images of FITC-HMC endocytosis by hemocytes after pre-treatment with 1, 3, 6, or 9 mg/ml of mannan. Scale bar = 2 μm. Nuclei are stained with Hoechst 33,342 (*blue*). Mean fluorescence intensity was quantified using ImageJ. *G*, microscopy images showing hemocytes that have endocytosed FITC-labeled HMC-Con and HMC-deMan. Scale bar = 2 μm. Nuclei are stained with Hoechst 33,342 (*blue*). Results are presented as mean ± SEM (n = 3). ∗*p* < 0.05, ∗∗*p* < 0.01, ∗∗∗*p* < 0.001 vs. control. Error bars represent S.E.

Building on our previous findings that post-translational modification (PTM) influence the antibacterial activity of penaeid shrimp hemocyanin (30, 31), we investigated whether acetylation, phosphorylation, and mannosylation affect its ability to enhance pathogen endocytosis by hemocytes. We generated deacetylated (HMC-deAc) (Fig. S4*A*), dephosphorylated (HMC-dePhos) (Fig. S4*B*), and low-mannose (HMC-deMan) (Fig. S4*C*) hemocyanin samples. Among these, the endocytosis of HMC-deMan was significantly reduced compared to the control (HMC-Con), HMC-deAc, and HMC-dePhos (Fig. 5*A*). However, all modified hemocyanin forms maintained strong binding to *V*. *parahaemolyticus*, *S*. *aureus*, and WSSV (Fig. S4, *D*–*F*).

Given that the mannose receptor (MR) has been implicated in hemocyanin endocytosis by mammalian dendritic cells and macrophages (25), we examined its role in shrimp hemocytes. Knockdown of MR significantly reduced hemocyanin uptake (Fig. 5, *B*–*D*). Since MR is a Ca^2+^-dependent glycan recognition receptor, we further assessed the effects of Ca^2+^ and mannan on hemocyanin endocytosis. While varying Ca^2+^ and mannan concentrations did not affect hemocyte viability or cellular activity (Fig. S4, *G*–*H*), Ca^2+^ induction significantly enhanced hemocyanin uptake (Fig. 5*E*), whereas increasing mannan concentrations significantly suppressed it (Fig. 5*F*). Additionally, the endocytosis of HMC-deMan was significantly lower than that of HMC-Con (Fig. 5*G*), confirming that hemocyanin mannosylation is crucial for enhanced endocytosis.

Since MR can mediate cellular uptake through clathrin-mediated endocytosis (32, 33), we further examined this pathway. Dual knockdown of MR and clathrin led to a significant decrease in hemocyanin uptake by hemocytes, both *in vivo* and *in vitro*, compared to individual knockdowns (Fig. S4, *K*–*L*), indicating that hemocyanin enters hemocytes *via* MR-clathrin-mediated endocytosis.

### Mannosylation enhances hemocyanin-mediated pathogen endocytosis by shrimp hemocytes

**Figure 6:**
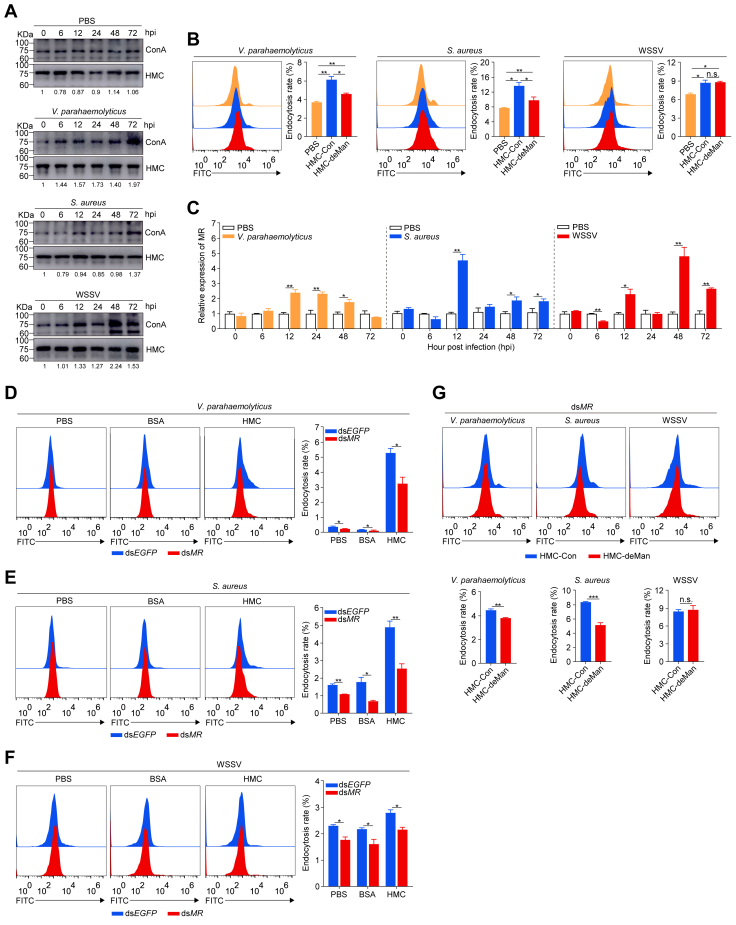
**Mannose receptor regulates hemocyanin-mediated microbial endocytosis**. *A*, Western blot analysis of plasma hemocyanin mannose modification at different time points post-infection with *V*. *parahaemolyticus*, *S*. *aureus*, and WSSV, with PBS as a control. *B*, relative endocytosis of FITC-labeled *V*. *parahaemolyticus*, *S*. *aureus*, and WSSV pre-incubated with HMC-Con, HMC-deMan, or PBS by shrimp hemocytes, as determined by flow cytometry. *C*, mRNA expression of *MR* in hemocytes at different time points (0, 6, 12, 24, 48, and 72 h) post-challenge with *V*. *parahaemolyticus*, *S*. *aureus*, and WSSV. *D-F*, relative endocytosis of FITC-labeled (*D*) *V*. *parahaemolyticus*, (*E*) *S*. *aureus*, and (*F*) WSSV by shrimp hemocytes following *MR* knockdown, as determined by flow cytometry. *G*, relative endocytosis of FITC-labeled *V*. *parahaemolyticus*, *S*. *aureus* and WSSV pre-incubated with HMC-Con or HMC-deMan by hemocytes following *MR* knockdown, as determined by flow cytometry. Results are presented as mean ± SEM (n = 3). ∗*p* < 0.05, ∗∗*p* < 0.01, ∗∗∗*p* < 0.001 vs. control. Error bars represent S.E. Immunoblots are representative of at least three independent experiments. BSA, bovine serum albumin; EGFP, enhanced *green* fluorescent protein; HMC, hemocyanin; HMC-Con, control hemocyanin; HMC-deMan, demannosylated hemocyanin; *MR*, mannose receptor.

To further investigate the role of hemocyanin mannosylation in pathogen endocytosis, we measured plasma levels of mannosylated hemocyanin in shrimp challenged with different microbial pathogens. Significantly elevated levels were detected following *V*. *parahaemolyticus*, *S*. *aureus*, and WSSV challenge compared with PBS control (Fig. 6*A*). When the effects of high-mannosylation (HMC-Con) and low-mannosylation (HMC-deMan) hemocyanin on pathogen uptake by hemocytes were analyzed, HMC-Con significantly enhanced (*p* < 0.05) the endocytosis of *V*. *parahaemolyticus* and *S*. *aureus* compared with HMC-deMan, but not WSSV (Fig. 6*B*). These results suggest that hemocyanin mannosylation is essential for bacterial pathogen binding and internalization by shrimp hemocytes.

Given the influence of global climate change on shrimp immune responses, we examined how temperature stress (24°C, 26°C, 28°C, and 30°C) affects hemocyanin mannosylation. Compared with the 24°C control, high-temperature stress significantly increased hemocyanin mannosylation (Fig. S5*A*) and enhanced its uptake by hemocytes (*p* < 0.05) (Fig. S5*B*). This suggests that temperature-induced hemocyanin modifications may modulate shrimp resistance to infections.

In shrimp aquaculture, ammonia nitrogen is a major environmental pollutant that affects hemocyanin’s structure and immune function (34, 35). Under ammonia nitrogen stress, hemocyanin mannosylation levels were significantly elevated (Fig. S5*C*), and hemocyte uptake of mannosylated hemocyanin was enhanced (*p* < 0.05) (Fig. S5*D*). Additionally, hemocyanin with higher mannosylation significantly increased the internalization of *V*. *parahaemolyticus*, *S*. *aureus*, and WSSV by hemocytes (*p* < 0.05) (Fig. S5*E*). These findings indicate that environmental stressors, such as elevated temperature and ammonia, regulate hemocyanin mannosylation, thereby enhancing hemocyte endocytosis and strengthening shrimp immune defenses against pathogens.

Since the mannose receptor (MR) functions as a pathogen pattern recognition receptor, we investigated whether it also serves as a receptor for hemocyanin during endocytosis. Following *V*. *parahaemolyticus*, *S*. *aureus*, and WSSV challenge, MR transcript levels were significantly upregulated (Fig. 6*C*). Conversely, MR knockdown significantly reduced the endocytosis of all three pathogens by hemocytes compared with the control (Fig. 6, *D*–*E*). Furthermore, bacteria pretreated with HMC-deMan showed significantly reduced uptake compared with HMC-Con after *MR* knockdown, whereas no significant difference was observed in WSSV endocytosis (Fig. 6*G*). These results highlight the crucial role of MR in mediating hemocyanin mannosylation, facilitating pathogen binding, and enhancing microbial clearance by shrimp hemocytes.

## Discussion

In arthropods and mollusks, the circulatory fluid, hemolymph, consists of hemocytes (cells) suspended in plasma, with hemocyanin as the predominant plasma protein. While over 90% of hemocyanin is found in the plasma, its intracellular levels in hemocytes are relatively low (27, 36). This suggests the presence of a regulatory mechanism that maintains this concentration gradient while allowing hemocyanin to enter hemocytes under specific conditions. In this study, we demonstrate that extracellular hemocyanin can enter shrimp hemocytes under normal physiological conditions, with uptake significantly enhanced under pathophysiological conditions, particularly upon stimulation with PAMPs, such as LPS, LTA, and PGN, as well as upon exposure to Gram-positive and Gram-negative bacteria or viruses. Our findings reveal that penaeid shrimp hemocytes internalize hemocyanin *via* multiple endocytic pathways, including phagocytosis, clathrin-mediated endocytosis, and macropinocytosis. Notably, we demonstrate that the mannose receptor (MR) facilitates hemocyanin uptake *via* clathrin-mediated endocytosis. Furthermore, we show that hemocyanin binding to microbial pathogens enhances their endocytosis and intracellular clearance, a process that is significantly influenced by hemocyanin mannosylation.

Eukaryotic cells internalize various molecules and particles, including nutrients, signaling factors, microbial pathogens, and other foreign objects, through endocytosis (37). In vertebrates, albumin, the most abundant plasma protein with minimal intracellular presence (38), is internalized by cells *via* clathrin-mediated endocytosis and micropinocytosis (39). Similarly, in crustaceans, hemocyanin, the major oxygen-transport protein and most abundant total protein hemolymph (40, 41), is found in significantly lower concentrations within shrimp hemocytes than in plasma (Fig. 1). Despite this concentration gradient, prior studies in the Chilean abalone (*Concholepas concholepas*) have shown that molluscan hemocytes can internalize hemocyanin (23). Consistent with this, we found that different macrophage-like cells, including shrimp hemocytes (*P*. *vannamei*), *Drosophila* S2 cells, and murine RAW264.7 macrophages, internalize *P*. *vannamei* hemocyanin through multiple endocytic pathways, with phagocytosis being the primary route (Fig. 2 and S2). Unlike bovine serum albumin (BSA), which served as an exogenous control, hemocyanin was preferentially and continuously internalized by shrimp hemocytes, indicating a physiologically relevant selective uptake mechanism. This preferential uptake likely plays a crucial role in enhancing pathogen endocytosis by shrimp hemocytes (Fig. 3). Even subtle differences in endocytic rates can have significant implications for pathogen clearance and immune modulation (Figs. 3 and 4). Moreover, the ability of both molluscan hemocyanin and vertebrate albumin to be internalized by cells underlies their utility as drug carriers or immunological adjuvants, highlighting their broader involvement in immune and physiological pathways (42, 43, 44, 45).

Beyond its primordial role in oxygen transport, shrimp hemocyanin also exhibits immune-related activities. Our previous studies have demonstrated functional differences among various *P*. *vannamei* hemocyanin subunits (46, 47, 48), which possess distinct structural and functional properties (49). Here, we show that recombinant hemocyanin proteins representing different subunit types are internalized at varying efficiencies by hemocytes (Fig. 1, *H*–*I*), suggesting that hemocyanin’s structural conformation influences its cellular uptake.

Invertebrates employ a range of antimicrobial immune responses, including phagocytosis, melanization, encapsulation, nodulation, lysis, RNA interference (RNAi)-mediated viral suppression, autophagy, and apoptosis (50). Among these, phagocytosis is particularly crucial for aquatic invertebrates such as crustaceans (51, 52). Our findings suggest that the internalization of hemocyanin from shrimp plasma into hemocytes plays a key role in antimicrobial defense, as hemocyanin significantly enhances the endocytosis of bacterial (*V*. *parahaemolyticus*, *V*. *alginolyticus*, *S*. *aureus*, *S*. *iniae*) and viral (WSSV) pathogens, thereby facilitating their intracellular clearance (Figs. 3 and 4). Notably, shrimp hemocytes internalized approximately 4 to 7% of pathogens bound to hemocyanin, a 2- to 3.5-fold increase compared to the ∼2% uptake rate of unbound pathogens by crustacean hemocytes reported in previous studies (53). While these absolute percentages may appear modest, the relative increase is substantial, indicating that hemocyanin-mediated enhancement of endocytosis is a physiologically meaningful immune mechanism. Furthermore, variations in hemocyanin’s ability to bind different bacterial species (Fig. 3*F*) (54), suggest that certain pathogens may exhibit a preference for specific hemocyanin subtypes, which could influence their uptake by hemocytes. This is consistent with previous studies in the red swamp crayfish (*Procambarus clarkii*), where hemocyanin and its derived peptides promoted bacterial phagocytosis by hemocytes (55).

Hemocyanin appears to function as an opsonin, binding to bacteria and viruses to enhance their recognition and uptake by hemocytes, with a particularly strong effect on bacterial endocytosis (Figs. 3 and 4). While hemocyanin binding also facilitated WSSV uptake and clearance, increasing hemocyanin concentrations did not further enhance viral endocytosis (Fig. 3*E*). This may be due to hemocyanin-mediated viral agglutination, which could inhibit viral attachment to and entry into host cells, a phenomenon previously observed in the black-lip abalone (*Haliotis rubra*) hemocyanin (56). Given that *Penaeus monodon* hemocyanin does not alter WSSV morphology or structure but instead attenuates viral replication (57), it is plausible that excess hemocyanin interferes with viral entry rather than promoting endocytosis. Consistent with this, our previous study in *P*. *vannamei* identified a hemocyanin-derived peptide (PvHcL48) that interacts with the WSSV VP28 protein, inhibiting viral replication (58).

Post-translational modifications (PTMs) can significantly influence the antimicrobial activity of hemocyanin. We recently reported that dephosphorylation at Thr517 and deacetylation at K481/K484 enhance its antibacterial activity (30, 31). In this study, we found that while mannosylation, deacetylation, and dephosphorylation all increased hemocyanin’s binding affinity to Gram-positive and Gram-negative bacteria as well as WSSV, mannosylation led to significantly greater endocytosis of hemocyanin by hemocytes during pathogen challenge compared to the other modifications. These differences suggest distinct mechanisms of antimicrobial action: whereas all three PTMs enhance hemocyanin-pathogen interactions, mannosylation promotes intracellular clearance *via* increased endocytosis, while dephosphorylation and deacetylation primarily facilitate extracellular pathogen clearance, resulting in lower hemocyanin internalization. Additionally, hemocyanin internalization appears to require structural modifications or specific cell surface receptors. Notably, molluscan hemocyanin is internalized by dendritic cells (DC) through mannose receptor (MR) and DC-specific ICAM-3-grabbing nonintegrin (DC-SIGN) (25), suggesting a conserved role for carbohydrate recognition in hemocyanin uptake.

The mannose receptor (MR) is a type I C-type lectin that binds carbohydrates in a calcium-dependent manner (59) and plays a critical role in glycoprotein homeostasis, pathogen recognition, endocytosis, and inflammation (60). In mammalian immune cells, MR also facilitates the internalization of molluscan hemocyanin (25). In the present study, we found that MR is essential for the mannosylation-dependent endocytosis of hemocyanin by shrimp hemocytes, occurring *via* clathrin-mediated endocytosis. Most importantly, bacterial and viral infections upregulated MR expression in hemocytes and increased the plasma levels of mannosylated hemocyanin (Fig. 6), highlighting MR’s role as a key pattern recognition receptor in penaeid shrimp. This suggests that MR-mediated hemocyanin uptake enhances immune defense by facilitating the endocytosis of microbial pathogens.

**Figure 7:**
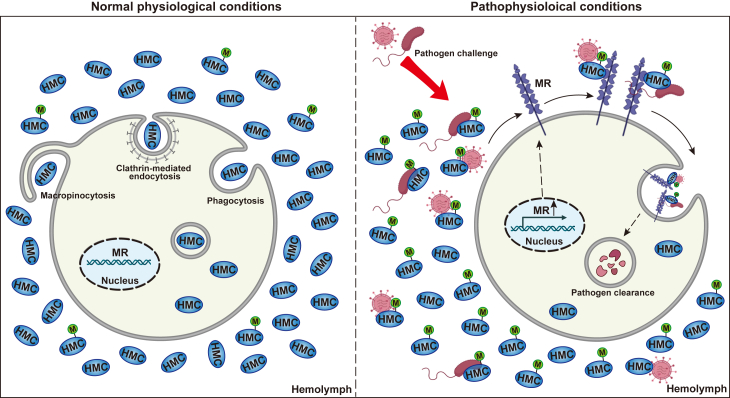
**Plasma hemocyanin enters hemocytes and enhances microbial endocytosis**. Under physiological conditions, shrimp hemocytes internalize extracellular plasma hemocyanin *via* phagocytosis, clathrin-mediated endocytosis, and macropinocytosis. However, under pathological conditions, hemocyte surface mannose receptors (MR) are upregulated, promoting mannose modification of hemocyanin. This enhances hemocyanin uptake and enables its interaction with microbial pathogens, facilitating their endocytosis for intracellular clearance.

Overall, our study demonstrates that during microbial infections in penaeid shrimp, hemocyanin undergoes post-translational modifications, such as mannosylation, to enhance its binding affinity to pathogens. This modification facilitates mannose receptor-mediated endocytosis by hemocytes, promoting intracellular pathogen clearance (Fig. 7). These findings highlight a previously underappreciated role of hemocyanin in shrimp immunity and suggest that PTM-driven modulation of hemocyanin function may be a critical mechanism for host defense against microbial infections.

## Experimental procedures

### Shrimp maintenance

Penaeid shrimp (*P*. *vannamei*, 5–8 g) were obtained from Shantou Huaxun Aquatic Product Corporation. Shrimp were acclimatized in air-circulating tanks containing artificial seawater (3–5 ppm salinity, 25 ± 2 °C) for 2 to 3 days. During acclimation, shrimp were fed daily with a commercial diet containing 35% protein (Tianma Science and Technology). All animal experiments were performed in accordance with the regulations and guidelines of the Animal Research and Ethics Committee of Shantou University.

### Cell culture and hemocyte isolation

Shrimp hemolymph was collected from healthy shrimp *via* the pericardial sinus using a sterile needle and syringe containing an equal volume of precooled anticoagulant (450 mM NaCl, 10 mM KCl, 10 mM Na_2_EDTA, 10 mM HEPES, pH 7.0). Hemocytes were isolated by centrifugation at 500×*g* for 10 min at 4 °C and resuspended in Insect-XPRESS medium (Lonza, Cat# 12–730Q) supplemented with 1% penicillin-streptomycin (Thermo Fisher Scientific, Cat# 15140122). Undispersed cells and debris were removed by filtration through a 35-μm nylon mesh. The cell suspension was adjusted to 1 × 10^6^ cells/ml and incubated at 28°C in a humidified incubator.

*Drosophila Schneider* two cells (S2 cells), a gift from Dr Haoyang Li (Sun Yat-sen University), were maintained in Schneider’s *Drosophila* medium (Thermo Fisher Scientific, Cat# 21720024) supplemented with 10% fetal bovine serum (FBS) (Thermo Fisher Scientific, Cat# A5669701) and 1% penicillin-streptomycin at 28 °C. RAW264.7 macrophages, a gift from Dr Chiju Wei (Shantou University, China), were cultured in DMEM (Thermo Fisher Scientific, Cat# 11965092) supplemented with 10% FBS and 1% penicillin-streptomycin at 37 °C in a 5% CO_2_ humidified incubator. *Mycoplasma* contamination was assessed using the MycoAlert *mycoplasma* detection kit (Lonza, Cat# LT07–418).

### Bacterial and viral strains

The bacterial strains *V*. *parahaemolyticus* (Marine Culture Collection of China, Cat# 1H00057), *V*. *alginolyticus*, *S*. *aureus* (Marine Culture Collection of China, Cat# 1A02584), and *S*. *iniae* were maintained in our laboratory. Bacteria from frozen stocks were cultured for 16 h at 37 °C with shaking at 200 rpm in Luria-Bertani medium and collected at OD_600_ = 0.6 for experiments. White spot syndrome virus (WSSV, China strain, GenBank: AF332093.3) was prepared from infected *P*. *clarkii* as previously described (61). Viral titers were determined by quantitative real-time PCR (qPCR) targeting the WSSV VP28 gene.

### Preparation of FITC-labeled and recombinant hemocyanin proteins

For endogenous hemocyanin purification, hemolymph samples were collected from three randomly selected shrimp and centrifuged at 800×*g* for 10 min at 4 °C to remove cells. The supernatant was further centrifuged at 20,000×*g* for 30 min at 4 °C to eliminate bacteria and debris. Purified hemocyanin was obtained using molecular sieve chromatography (Solarbio, Cat# S9160), and its concentration was determined using a BCA protein assay kit (Genstar Biotechnology, Cat# E162–01). Protein purity was verified by SDS-PAGE and the Bradford assay. The purified hemocyanin was labeled with fluorescein isothiocyanate (FITC) using a Quick FITC Antibody Labeling Kit (Meilunbio, Cat# MA0362). Bovine serum albumin (BSA, Genstar Biotechnology, Cat# E162–01) was similarly labeled as a control. FITC-labeled hemocyanin (HMC-FITC) and FITC-labeled BSA (BSA-FITC) were stored at −80 °C.

For recombinant hemocyanin expression, cDNA sequences of hemocyanin subunit 1 (UniProtKB: A0A059TEW9) and hemocyanin subunit 2 (UniProtKB: A0A1Y0DT76) were cloned into the pIZ/V5-His vector (Thermo Fisher Scientific, Cat# V801001). S2 cells were seeded into 6-well plates and transfected with plasmids pIZ-HMC subunit 1-EGFP-Flag, pIZ-HMC subunit 2-EGFP-Flag, or pIZ-EGFP-Flag using HD transfection reagent (Promega, Cat# E2311). After 48 h, cells were lysed in buffer (25 mM HEPES, 150 mM NaCl, 1% Triton X-100, 1 mM Na_2_EDTA, pH 7.4) supplemented with a protease inhibitor cocktail (MedChemExpress, Cat# HY-K0010) and PMSF (Beyotime Biotechnology, Cat# ST505). Lysates were centrifuged at 20,000×*g* for 10 min at 4 °C, and the supernatant was incubated with anti-Flag magnetic beads (MedChemExpress, Cat# HY-K0207) at 4 °C for 30 min. Beads were washed five times with PBS and eluted with 1 mg/ml 3 × Flag peptide (50 mM Tris, 0.15 M NaCl, pH 7.4). Proteins were dialyzed in 0.01 M PBS using 30-kDa filter devices and concentrated by centrifugation (12,000×*g* for 2 min at 4 °C). Purified proteins were stored at −40 °C.

For mannose-modified hemocyanin, purified hemocyanin was treated with α1-2,3,6-mannosidase (New England BioLabs, Cat# P0768S) to generate low-mannose hemocyanin. Dephosphorylated and deacetylated hemocyanin variants were prepared by incubating purified hemocyanin with protein phosphatase 2A (PP2A) and histone deacetylase 3 (HDAC3), respectively, *in vitro*. Control hemocyanin samples were treated with 0.01 M PBS. The control (HMC-Con), low-mannose (HMC-deMan), low-phosphorylated (HMC-dePhos), and low-acetylated hemocyanin (HMC-deAc) samples were FITC-labeled as described above.

### RNA interference

Double-stranded RNA (dsRNA) was synthesized to knock down *P*. *vannamei* MR (*GenBank*: XP_027231195.1) or the non-endogenous *EGFP* (*Gene ID*: U55762.1) using Primer Premier five software (Table 1). The dsRNA was generated using the T7 High Yield RNA Synthesis Kit (Cat# E2040S, New England Biolabs), and its integrity was verified *via* 1% agarose gel electrophoresis. Before use, dsRNA was diluted to 20 μg/ml in RNase-free water and stored at −80 °C. Shrimp were divided into two groups (20 shrimp each): the experimental group received 100 μl of 20 μg/ml dsMR, while the control group received an equivalent amount of dsEGFP. After 24 h, hemocytes were collected for further analysis.

**Table 1:** Sequences of primers and dsRNAs used in this study

Name	Sequence (5′-3′)
VP28-F	ATGGATCTTTTCTTTCACTCTTTC
VP28-R	TTACTCGGTCTCAGTGCCAG
qVa-F (*groEL* gene)	GATTCGGTGAAGAAGAGATGATCTC
qVa-R (*groEL* gene)	TCTTCGTTGTCACCCGTTAGGTGA
qSA-F (*nuc* gene)	TCGCTTGCTATGATTGTGG
qSA-R (*nuc* gene)	ACATACGCCAATGTTCTACC
qSI-F (*lldP* gene)	ACACAGGTGAGCACGCTAAA
qSI-R (*lldP* gene)	CGTCACCATCGTCTTGGTCA
qVp-F (*tdh* gene)	GTAAAGGTCTCTGACTTTTGGAC
qVp-R (*tdh* gene)	TGGAATATGAACCTTCATCTTCACC
q*PvMR* -F	TGTTCAGCCCGATCCACAT
q*PvMR*-R	GACGCCAACTGCCTCATCC
q*Pv*clathrin-F	ATTTGATAGAGTTCCGTCG
q*Pv*clathrin-R	GCAGGTCGTAGCAGTGGA
dsEGFP-F	CGTAAACGGCCACAAGTT
dsEGFP-R	TTCACCTTGA TGCCGTTC
dsEGFP-F T7	GGATCCTAATACGACTCACTATAGGCGTAAACGGCCACAAGTT
dsEGFP-R T7	GGATCCTAATACGACTCACTATAGGTTCACCTTGATGCCGTTC
ds*PvMR*-F	AAGAACGAGCCCAACACCAA
ds*PvMR*-R	GTAGTCGAAGCCAGTGCCAT
ds*PvMR*-F T7	GGATCCTAATACGACTCACTATAGGAAGAACGAGCCCAAC ACCAA
ds*PvMR*-R T7	GGATCCTAATACGACTCACTATAGGGTAGTCGAAGCCAGT GCCAT
siNon-F	UUCUCCGAACGUGUCACGUTT
siNon-R	ACGUGACACGUUCGGAGAATT
si*Pv*MR-F	GAGCUCUGUACCGAGAAGATT
si*Pv*MR-R	UCUUCUCGGUACAGAGCUCTT
si*Pv*clathrin-F	GGUUAUGUUCACCUCUAUGTT
si*Pv*clathrin-R	CAUAGAGGUGAACAUAACCTT
*PvEF*1a-F	TATGCTCCTTTTGGACGTTTTGC
*PvEF*1a-R	CCTTTTCTGCGGCCTTGGTAG

### Endocytosis assays

To assess hemocyanin uptake by hemocytes *in vitro*, hemocytes (1 × 10^6^ cells/ml) were incubated with 350 nM of FITC-labeled hemocyanin (HMC-FITC) or bovine serum albumin (BSA-FITC) and 400 nM of EGFP, recombinant hemocyanin subunit 1 (rHMC-Sub1), or recombinant hemocyanin subunit 2 (rHMC-Sub2) at 28 °C for 2 h in the dark. Samples were washed three times with culture medium, followed by a 20-min incubation with 10 μg/ml Hoechst 33,342 (Cat# 40731ES10, Yeasen Biotechnology).

For *in vivo* endocytosis, shrimp were divided into eight groups (20 shrimp each) and injected with 100 μl of HMC-FITC or BSA-FITC at final concentrations of 0.75, 1.5, 3, or 6 μM into the second abdominal segment. After 2 h, hemolymph was collected from three randomly selected shrimp per group and treated with 0.04% trypan blue to quench extracellular fluorescence.

To evaluate the impact of pathogen-associated molecular patterns (PAMPs) on hemocyanin uptake *in vitro*, hemocytes were pre-treated with 10 ng/ml lipopolysaccharides (LPS, Cat# L2630, Sigma-Aldrich), 100 ng/ml lipoteichoic acid (LTA, Cat# L3140, Sigma-Aldrich), or 10 ng/ml peptidoglycan (PGN, Cat# D1662, Sigma-Aldrich) for 30 min before incubation with 350 nM of HMC-FITC or BSA-FITC for 2 h in the dark. Similarly, for *in vivo* analysis, shrimp were divided into eight groups (20 shrimp each) and injected with 20 μg of LPS, 10 μg of LTA, or 10 μg of PGN in 100 μl PBS. Control shrimp received an equivalent volume of PBS. After 24 h, shrimp were injected with 6 μM of HMC-FITC or BSA-FITC, and hemolymph was collected 2 h post-injection to measure endocytosis rates.

### Endocytosis inhibition, mannan treatment, and calcium induction

To investigate the mechanisms of hemocyanin internalization, hemocytes were treated with specific endocytosis inhibitors for 30 min, including cytochalasin B (Cat# HY-16928, MedChemExpress), chlorpromazine (Cat# HY-B0407 A, MedChemExpress), amiloride hydrochloride (Cat# HY-B0285 A, MedChemExpress), genistein (Cat# HY-14596, MedChemExpress), and methyl-β-cyclodextrin (Cat# HY-101461, MedChemExpress). The inhibitors were used at the following concentrations: cytochalasin B (Cyt B, at 2 μg/ml), chlorpromazine (CPZ, at 100 μM), amiloride hydrochloride (AMR, at 100 μM), genistein (GEN, at 50 μM), and methyl-β-cyclodextrin (MβCD, at 50 μM).

For calcium induction, hemocytes were treated with 4, 6, or 8 mM Ca^2+^ (CaCl_2_) or 8 mM Ca^2+^ and 4 mM EDTA for 30 min. Mannan competition assays were performed by incubating hemocytes with 1, 3, 6, or 9 mg/ml of mannan (Cat# M7504, Sigma-Aldrich) for 30 min. Cells from all treatment groups were subsequently incubated with 350 nM HMC-FITC or BSA-FITC for 2 h in the dark. Hemocyanin uptake was then analyzed *via* laser scanning confocal microscopy and flow cytometry.

### Phagocytic activity

To assess the role of hemocyanin in hemocyte phagocytosis under normal and pathogen-challenged conditions, bacteria (*V*. *parahaemolyticus*, *V*. *alginolyticus*, *S*. *aureus*, *S*. *iniae*) and virus (WSSV) were labeled with FITC or DiL (Cat# C1991S, Beyotime Biotechnology) following a previously described method (62). Briefly, 10 μl of 10 mg/ml FITC or DiL was added to 1 ml of bacterial suspension and incubated at room temperature for 1 h. For WSSV labeling, 20 μl of a 1 × 10^7^ copies/μl viral suspension (in 20 mM Tris-HCl, 2 mM MgCl_2_, 150 mM NaCl, pH 7.5, 0.45 μm membrane-filtered) was mixed with 80 μl of 100 μg/ml FITC and incubated at room temperature for 1 h. Labeled bacteria and WSSV were washed three times with sterile PBS and TMN buffer, respectively, before storage at −80 °C.

The FITC-labeled bacteria and virus were pre-incubated with HMC or BSA at 14, 70, or 350 nM concentrations or with sterile PBS (control) for 30 min. For *in vitro* phagocytosis assays, hemocytes were co-incubated with FITC-labeled bacteria at a 1:5 hemocyte-to-bacteria ratio or with WSSV at a multiplicity of infection (MOI) of 10 for 2 h in the dark, followed by three washes with culture medium.

For *in vivo* phagocytosis assays, shrimp were injected with 100 μl of 1 × 10^9^ CFU/ml of *V*. *parahaemolyticus* or *S*. *aureus* pre-incubated with 350 nM HMC-FITC, BSA-FITC, or PBS. Alternatively, shrimp were injected with 100 μl of 1 × 10^5^ copies of WSSV pre-incubated with 70 nM HMC-FITC, BSA-FITC, or PBS. Hemolymph was collected from 25 shrimp per group at 2 h post-injection, and phagocytic rates were analyzed *via* flow cytometry (FCM).

### Pathogen treatment and binding assays

To assess hemocyanin binding to bacteria and viruses, 200 μl of purified hemocyanin (100 μg/ml) was mixed with 800 μl of bacterial suspensions (1 × 10^7^ CFU/ml) of *V*. *parahaemolyticus*, *V*. *alginolyticus*, *S*. *aureus*, or *S*. *iniae*. The mixtures were incubated at 37 °C for 30 min. For viral binding assays, 50 μl of *White Spot Syndrome Virus* (WSSV) (2 × 10^6^ copies/μl) was incubated with 200 μl of purified hemocyanin (100 μg/ml) for 30 min at room temperature. Samples were washed three times with 0.01 M PBS (pH 7.4) and resuspended in 20 μl of 5 × SDS loading buffer (42 mM Tris-HCl, 10% glycerol, 2.3% SDS, 5% 2-ME, and 0.002% bromophenol blue). The mixtures were boiled at 98 °C in a water bath for 15 min before SDS-PAGE and Western blot analyses were performed in replicates.

To evaluate pathogen-induced post-translational modifications, specifically the mannose modification of hemocyanin, shrimp were injected with 100 μl of bacterial suspensions (1 × 10^5^ CFU of *V*. *parahaemolyticus*, *V*. *alginolyticus*, *S*. *aureus*, or *S*. *iniae*) or viral suspensions (1 × 10^5^ copies of WSSV). Control shrimps were injected with an equal volume of 0.01 M PBS. Plasma and hemocytes were collected at 0, 6, 12, 24, 48, and 72 h post-injection and analyzed by Western blot using Concanavalin A (Cat# B-1005–5, Vector Laboratories) (1:5000 dilution) to detect mannose modification. Additionally, quantitative PCR (qPCR) was performed to determine the expression levels of the mannose receptor (MR) using gene-specific primers (Table 1).

To examine the effect of hemocyanin endocytosis on bacterial and viral loads in shrimp hemocytes, 100 μl of WSSV (1 × 10^5^ copies) was pre-incubated with 70 nM of FITC-labeled hemocyanin (HMC-FITC) or BSA-FITC. Similarly, 100 μl of bacterial suspensions (1 × 10^5^ CFU) was pre-incubated with 350 nM of HMC-FITC or BSA-FITC before injection into shrimp (n = 30 per group). Hemolymph was collected from 25 shrimp per group at 24 h post-injection, and total DNA was extracted using the TIANamp Marine Animals DNA Kit (Cat# DP324, TransGen Biotechnology). Bacterial loads and WSSV copies were quantified using qPCR with specific primers (Table 1).

### Flow cytometry and laser scanning confocal microscopy analysis

Flow cytometry was performed using an Accuri C6 Plus Flow Cytometer (BD Biosciences). For each sample, 10,000 events were recorded based on forward scatter (FSC) and side scatter (SSC) characteristics and analyzed using FCS Express (De Novo Software) and FlowJo v10.6.2 (BD Biosciences). Internalized FITC-labeled proteins were quantified using the FL1 channel, while internalized DiL-labeled pathogens were analyzed using the FL2 channel.

To determine protein localization within hemocytes, samples were examined using a Zeiss LSM 880 laser scanning confocal microscope (Carl Zeiss AG) at × 63 magnification. Images were processed and merged using ZEN software (Carl Zeiss AG) and ImageJ v1.46 (National Institutes of Health).

### Total RNA extraction, cDNA synthesis, and real-time PCR analysis

Total RNA was extracted from shrimp hemocytes using TRIzol reagent (Cat# 15596018, Invitrogen) following the manufacturer’s protocol. RNA concentration was measured using a NanoDrop 2000 spectrophotometer (NanoDrop Technologies), and RNA integrity was verified by 1% agarose gel electrophoresis and A260/280 ratio analysis. Only high-quality RNA was used for subsequent analysis.

First-strand cDNA synthesis was performed using 1.0 μg of total RNA and the One-Step gDNA Removal and cDNA Synthesis SuperMix kit (Cat# AT311, TransGen Biotech). Real-time PCR reactions were conducted using a 10 μl 2 × RealStar Green Power Mix (Cat# A311, GenStar Biotechnology, Beijing, China), 1 μl cDNA template (10 ng/ml), 1 μl each of forward and reverse primers (10 mM), and 7 μl RNase-free water. The thermocycling conditions were pre-denaturation at 95 °C for 5 min, 40 cycles of denaturation at 95 °C for 15 s, annealing at 60 °C for 30 s, extension at 72 °C for 30 s, and final extension at 72 °C for 10 min.

### Hemocytes and plasma protein levels, SDS-PAGE, and Western blot analysis

Hemolymph samples were collected from three shrimp, and plasma and cell lysates were prepared as described previously (30). Samples were either undiluted or serially diluted (0 × , 2 × , 4 × , 6 × , and 18 × ) with 0.01 M PBS before being mixed with 5 × SDS loading buffer and boiled for 10 min. Proteins were separated on 10% SDS-PAGE gels and transferred onto polyvinylidene difluoride (PVDF) membranes (Cat# IPVH00010, Millipore) using a Mini Trans-Blot Electrophoretic Transfer Cell (Bio-Rad). Membranes were blocked with 5% skimmed milk in TBST buffer (20 mM Tris, 150 mM NaCl, 0.1% Tween-20, pH 7.6) for 1 h at room temperature, then incubated overnight at 4 °C (or for 2 h at room temperature) with an in-house anti-hemocyanin antibody (1:3000 dilution). After washing three times with TBST buffer (15 min each), membranes were incubated for 1 h at room temperature with HRP-conjugated rabbit secondary antibody (Cat# 31460, ThermoFisher Scientific) at a 1:5000 dilution. Chemiluminescent signals were detected using an ECL reagent (Cat# WBLUF0100, Millipore), and images were captured with a GE Amersham Imager 600 (GE Healthcare). Gels and blots were quantitatively analyzed using ImageJ v1.46 (National Institutes of Health).

Hemocyanin concentrations in plasma and hemocytes were determined spectrophotometrically as described previously (63). Briefly, the absorbance of 300 μl of cell lysates and 20-fold diluted plasma was measured at 335 nm, and the concentration of hemocyanin was calculated using the following formula.

E335(mg/ml) = 2.3 × OD335 nm

Total protein concentrations were measured using a BCA protein assay kit, and the percentage of hemocyanin was calculated as:

Hemocyanin (%) = [Hemocyanin concentration (mg/ml)/Total protein concentration (mg/ml)] × 100.

### Statistical analysis

Data are presented as mean ± SD. Statistical significance was determined using Student’s *t* test or one-way ANOVA with GraphPad Prism 9 and Microsoft Excel 2021. Statistical significance was considered at *p* < 0.05.

## Data availability

All data presented are contained within the manuscript.

## Supporting information

This article contains supporting information.

## Conflict of interest

The authors declare that they have no conflicts of interest with the contents of this article.
