# Supplementary material for: Mannose-modified hemocyanin enhances pathogen endocytosis by crustacean hemocytes
Source: J Biol Chem. 2025 May 28;301(7):110304. doi: 10.1016/j.jbc.2025.110304 (PMC12221358; doi:10.1016/j.jbc.2025.110304)
Supplement: Supplementary Material [file mmc1.pdf]

Supporting information for

Mannose-modified hemocyanin enhances pathogen endocytosis by crustacean hemocytes

Jiaxi Li[#1], Jude Juventus Aweya[#1,3,4], Mingming Zhao[1], Yongzhen Zhao[2], Zhongyang Lin[1], Xiuli Chen[2], Zhihong Zheng[1], Pengfei Li[5,6], Defu Yao[1]*, Yueling Zhang[1]*

*Address correspondence to: Emails: zhangyl@stu.edu.cn and dfyao@stu.edu.cn

This PDF file includes:

Figs. S1 to S5

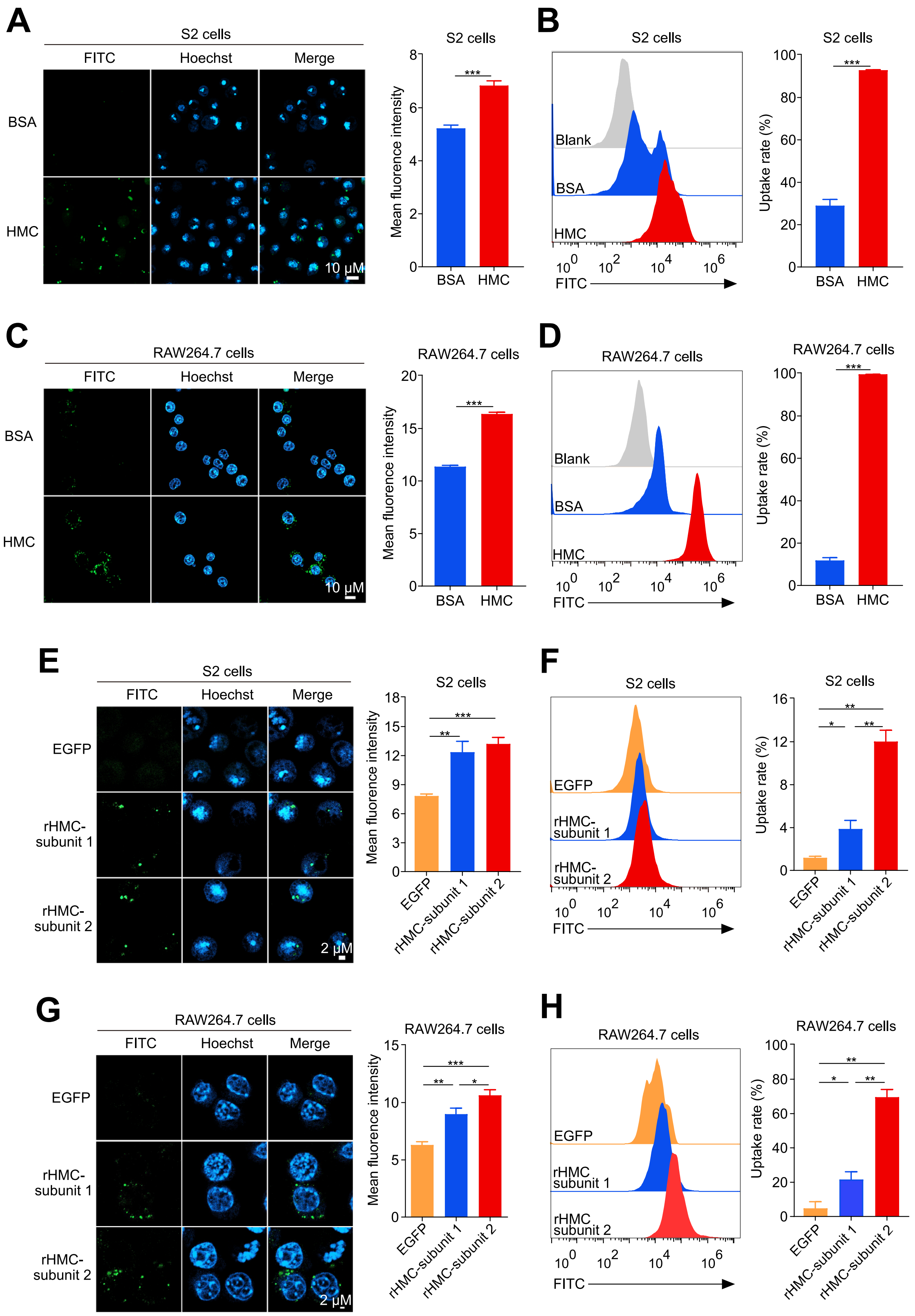
A
S2 cells
FITC
Hoechst
Merge
BSA
HMC
10 μM
Mean fluorence intensity
***
BSA
HMC
B
S2 cells
Blank
BSA
HMC
FITC
Uptake rate (%)
***
C
RAW264.7 cells
FITC
Hoechst
Merge
BSA
HMC
10 μM
Mean fluorence intensity
***
D
RAW264.7 cells
Blank
BSA
HMC
FITC
Uptake rate (%)
***
E
S2 cells
FITC
Hoechst
Merge
EGFP
rHMC-subunit 1
rHMC-subunit 2
2 μM
Mean fluorence intensity
***
**
F
S2 cells
EGFP
rHMC-subunit 1
rHMC-subunit 2
FITC
Uptake rate (%)
**
*
**
G
RAW264.7 cells
FITC
Hoechst
Merge
EGFP
rHMC-subunit 1
rHMC-subunit 2
2 μM
Mean fluorence intensity
***
**
*
H
RAW264.7 cells
EGFP
rHMC subunit 1
rHMC subunit 2
FITC
Uptake rate (%)
**
*
**

Fig. S1. Hemocyanin is internalized by S2 cells and RAW264.7 cells. (A-B) Laser confocal microscope and flow cytometry data showing the endocytosis of FITC-labeled hemocyanin (HMC) and BSA by S2 cells. Scale bar = 10 μm. Cell nuclei were stained with hoechst 33342 (blue). Mean fluorescence intensity of FITC-labeled HMC and BSA in S2 cells quantified using Image J software. Uptake rates of FITC-labeled HMC and BSA by S2 cells quantified using FlowJo software. (C-D) Laser confocal microscope and flow cytometry data showing the endocytosis of FITC-labeled hemocyanin (HMC) and BSA by RAW264.7 cells. Scale bar = 10 μm. Cell nuclei were stained with hoechst 33342 (blue). Mean fluorescence intensity of FITC-labeled HMC and BSA in RAW264.7 cells quantified using Image J software. Uptake rates of FITC-labeled HMC and BSA by RAW264.7 cells quantified using FlowJo software. (E-F) Laser confocal microscope and flow cytometry data showing the endocytosis of EGFP, rHMC-subunit 1, and rHMC-subunit 2 by S2 cells. Scale bar = 10 μm. Cell nuclei were stained with hoechst 33342 (blue). Mean fluorescence intensity of EGFP, rHMC-subunit 1, and rHMC-subunit 2 in S2 cells quantified using Image J software. Uptake rates of EGFP, rHMC-subunit 1 and rHMC-subunit 2 by S2 cells quantified using FlowJo software. (G-H) Laser confocal microscope and flow cytometry data showing the endocytosis of EGFP, rHMC-subunit 1, and rHMC-subunit 2 by RAW264.7 cells. Scale bar = 10 μm. Cell nuclei were stained with hoechst 33342 (blue). Mean fluorescence intensity of EGFP, rHMC-subunit 1, and rHMC-subunit 2 in RAW264.7 cells quantified using Image J software. Uptake rates of EGFP, rHMC-subunit 1 and rHMC-subunit 2 by RAW264.7 cells quantified using FlowJo software. Results reported as mean ± SEM (n = 3). The immunoblots images shown are representative of at least t**hree** independent experiments. Statistical significance was determined using Student's t-test (*$p < 0.05$, **$p < 0.01$, ***$p < 0.001$). Error bars represent S.E.

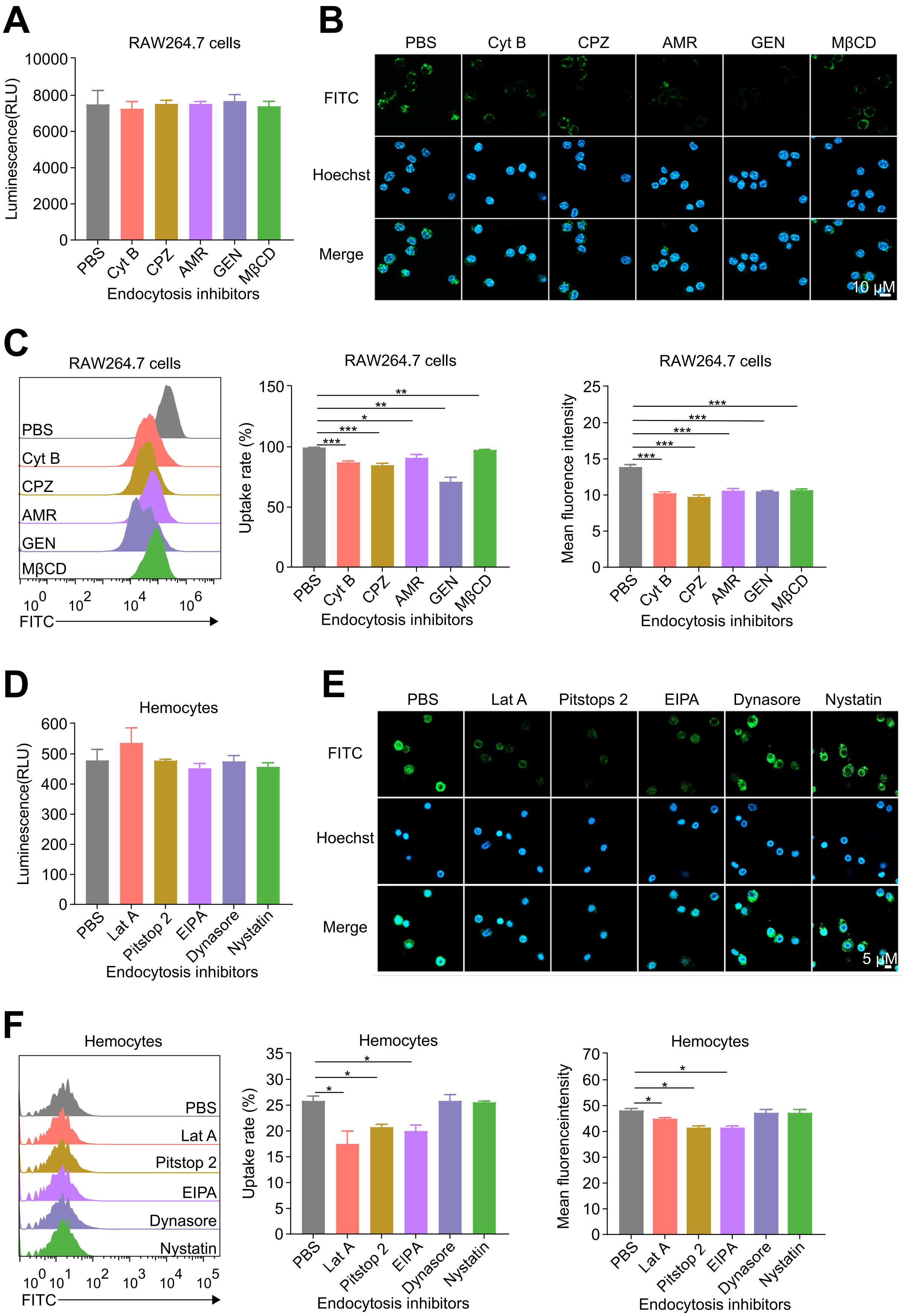

A
RAW264.7 cells
Luminescence(RLU)
10000
8000
6000
4000
2000
0
PBS
Cyt B
CPZ
AMR
GEN
MβCD
Endocytosis inhibitors
B
PBS
Cyt B
CPZ
AMR
GEN
MβCD
FITC
Hoechst
Merge
10 μM
C
RAW264.7 cells
PBS
Cyt B
CPZ
AMR
GEN
MβCD
FITC
RAW264.7 cells
Uptake rate (%)
Endocytosis inhibitors
RAW264.7 cells
Mean fluorence intensity
Endocytosis inhibitors
D
Hemocytes
Luminescence(RLU)
PBS
Lat A
Pitstop 2
EIPA
Dynasore
Nystatin
Endocytosis inhibitors
E
PBS
Lat A
Pitstops 2
EIPA
Dynasore
Nystatin
FITC
Hoechst
Merge
5 μM
F
Hemocytes
PBS
Lat A
Pitstop 2
EIPA
Dynasore
Nystatin
FITC
Hemocytes
Uptake rate (%)
Endocytosis inhibitors
Hemocytes
Mean fluorenceintensity
Endocytosis inhibitors

Fig. S2. Endocytosis of hemocyanin by RAW264.7 cells is mediated by specific endocytic pathways. (A) Effects of five endocytosis inhibitors on shrimp hemocytes cellular activity. Cells were treated with cytochalasin B (Cyt B), chlorpromazine (CPZ), amiloride (AMR), genistein (GEN), and methyl-β-cyclodextrin (MβCD). (B) Laser confocal microscopy images of the endocytosis and mean fluorescence intensity of FITC-labeled HMC by RAW264.7 cells treated with five types of endocytosis inhibitors. Scale bar = 10 μm. Nuclei were stained with hoechst 33342 (blue). (C) Flow cytometry data showing the endocytosis and uptake rates of FITC-labeled hemocyanin (HMC) by RAW264.7 cells treated with five types of endocytosis inhibitors. (D) Effects of five endocytosis inhibitors on shrimp hemocytes cellular activity. Cells were treated with latrunculin A (Lat A), pitstop 2, ethylisopropylamiloride (EIPA), dynasore, and nystatin. (E) Laser confocal microscopy images of the endocytosis and mean fluorescence intensity of FITC-labeled HMC by hemocytes treated with five types of endocytosis inhibitors. Scale bar = 10 μm. Nuclei were stained with hoechst 33342 (blue). (F) Flow cytometry data showing the endocytosis and uptake rates of FITC-labeled hemocyanin (HMC) by hemocytes treated with five types of endocytosis inhibitors. Results reported as mean ± SEM (n = 3). The immunoblots images shown are representative of at least three independent experiments. Statistical significance was determined using Student's t-test (*$p < 0.05$, **$p < 0.01$, ***$p < 0.001$). Error bars represent S.E.

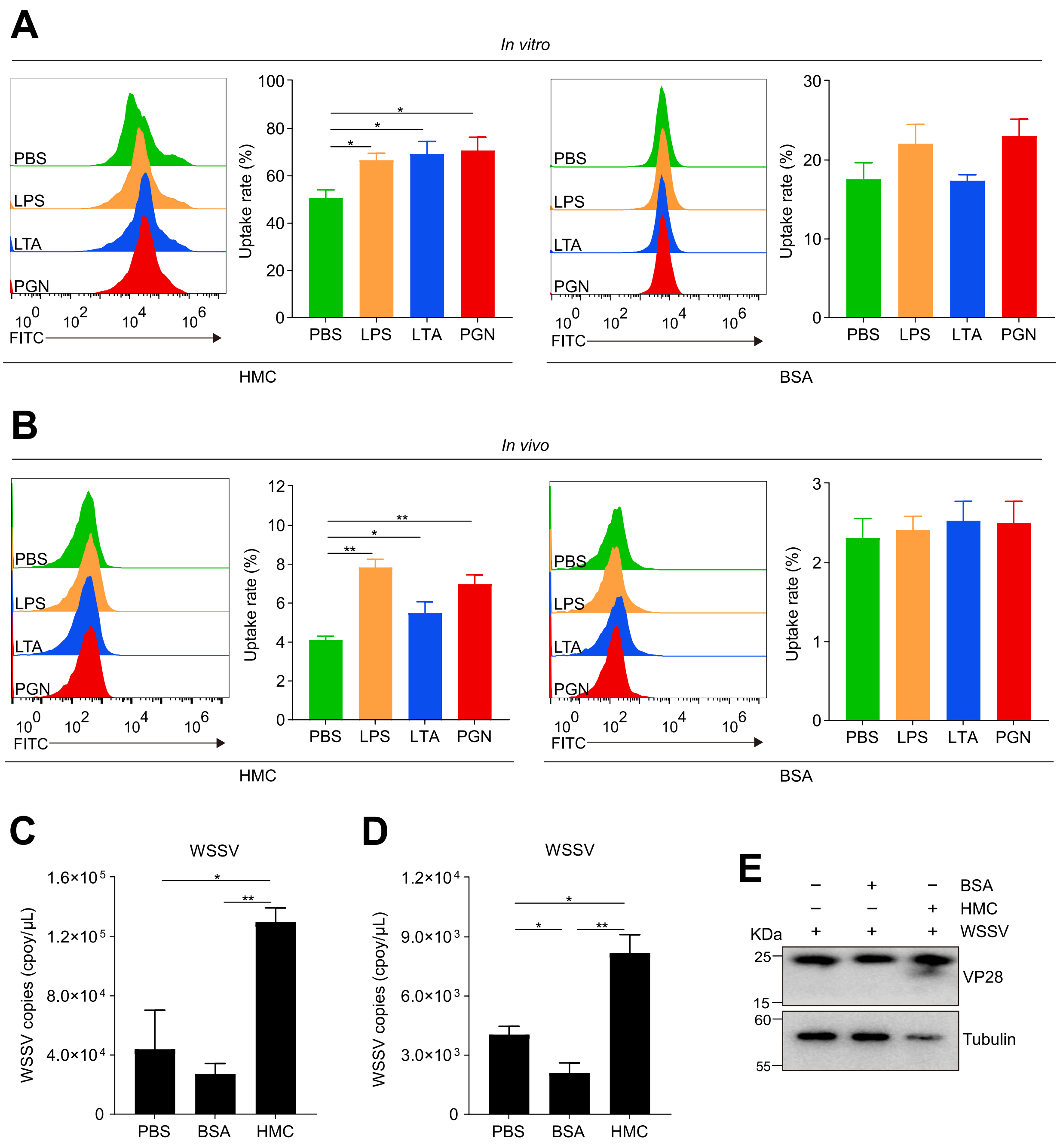

Fig. S3. WSSV treated with hemocyanin enhances phagocytic activity of hemocytes. (A) Flow cytometry data showing the endocytosis and uptake rates of FITC-labeled hemocyanin (HMC) and BSA by hemocytes treated in vitro with LPS (10 ng/ml), LTA (100 ng/ml), and PGN (20 ng/ml). (B) Flow cytometry data showing the endocytosis and uptake rates of FITC-labeled HMC and BSA by hemocytes in vivo after shrimp were challenged with LPS (20 μg/ml), LTA (10 μg/ml), and PGN (10 μg/ml) for 24 h before being injected 6 μM of FITC-labeled HMC or BSA for 2 h. (C) WSSV copies in hemocytes after challenging shrimp with WSSV pre-incubated with 70 nM HMC or BSA for 30 min. As a control, WSSV was treated with PBS. The WSSV envelope protein VP28 gene was an indicator of virus copies. (D) WSSV copies in hemocytes after WSSV were pre-incubated with 70 nM of HMC or BSA for 1 h in vitro. (E) WSSV VP28 protein expression in shrimp hemocytes after WSSV was pre-incubated with HMC or BSA

before being incubated with hemocytes. Statistical significance was determined using Student's t-test (*$p < 0.05$, **$p < 0.01$, ***$p < 0.001$). Error bars represent S.E.

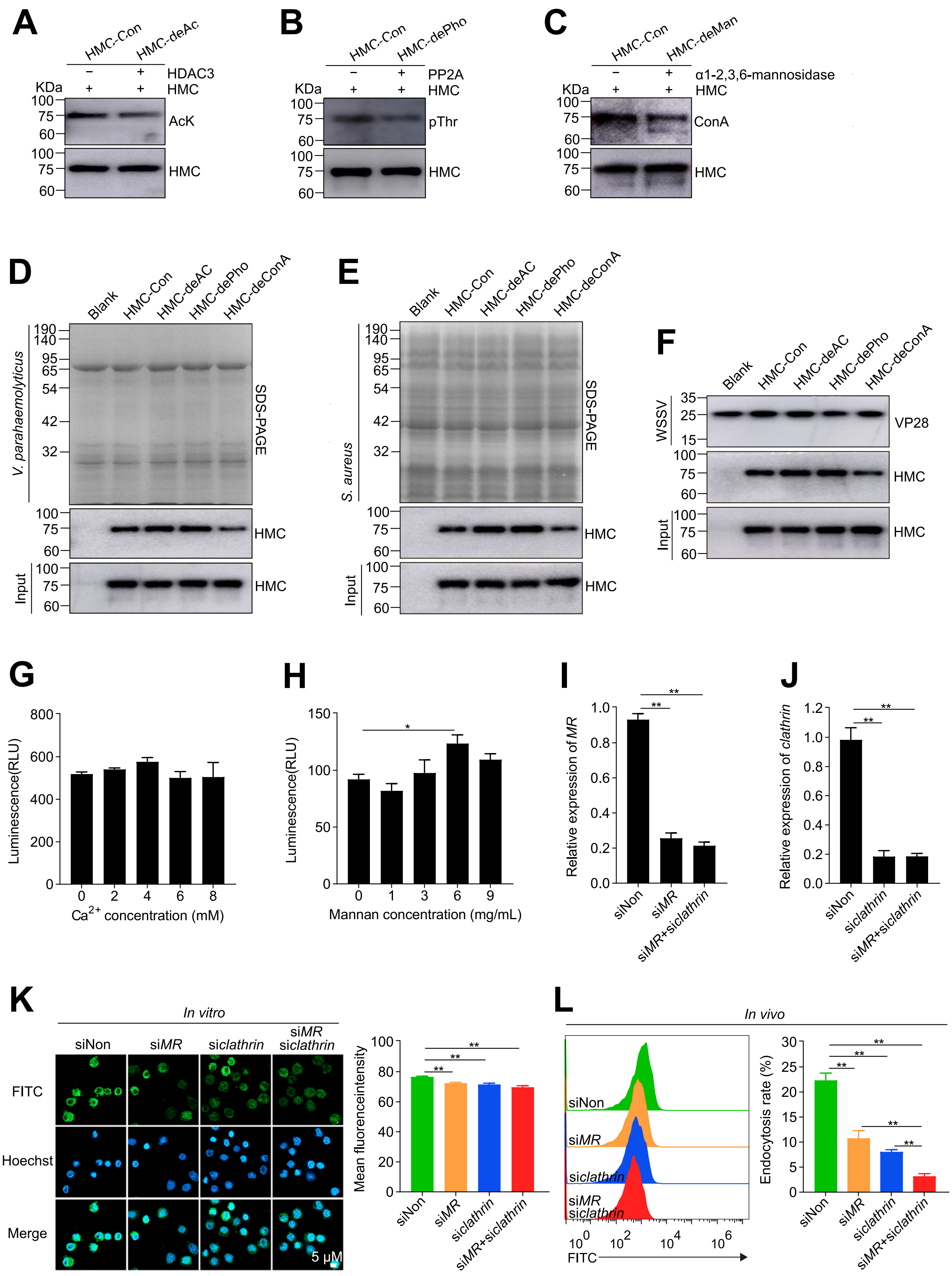

Fig. S4. Effects of Ca^{2+} and mannan on the cellular activity of hemocytes. (A) Low acetylation modified hemocyanin (HMC-deAc) and normal hemocyanin (HMC-Con) treated with histone

deacetylase 3 (HDAC3) and probed with acetyl-Lys antibody (Ack). (B) Low-threonine phosphorylated hemocyanin (HMC-dePhos) and normal hemocyanin (HMC-Con) treated with protein phosphatase 2A(PP2A) and probed with phospho-Thr antibody (pThr). (C) Low-mannose modified hemocyanin (HMC-deMan) and HMC-Con as a control treated with α1-2,3,6 mannosidase and detected using concanavalin A (ConA). Binding of HMC-Con, HMC-deAc, HMC-dePhos, and HMC-deMan with (D) *V. parahaemolyticus*, (E) *S. aureus*, and (F) WSSV. (G) Effects of different concentrations of Ca^{2+} (2, 4, 6, and 8 mM) on the cellular activity of hemocytes. (H) Effects of different concentrations of mannan (1, 3, 6, and 9 mg/mL) on the cellular activity of hemocytes. (I) Real-time PCR detection of MR gene interference efficiency in hemocytes. (J) Real-time PCR detection of clathrin gene interference efficiency in hemocytes. (K) Laser confocal microscopy images of the endocytosis and mean fluorescence intensity of FITC-labeled HMC by hemocytes with siNon-, siMR, siclathrin and siMR+siclathrin-knockdown. Scale bar = 5 μm. Nuclei were stained with hoechst 33342 (blue). (L) Flow cytometry data showing the endocytosis and uptake rates of FITC-labeled hemocyanin (HMC) by hemocytes under depletion of Non, MR, clathrin and both MR and clathrin. Statistical significance was determined using Student’ s t-test (*$p < 0.05$, **$p < 0.01$, ***$p < 0.001$). Error bars represent S.E.

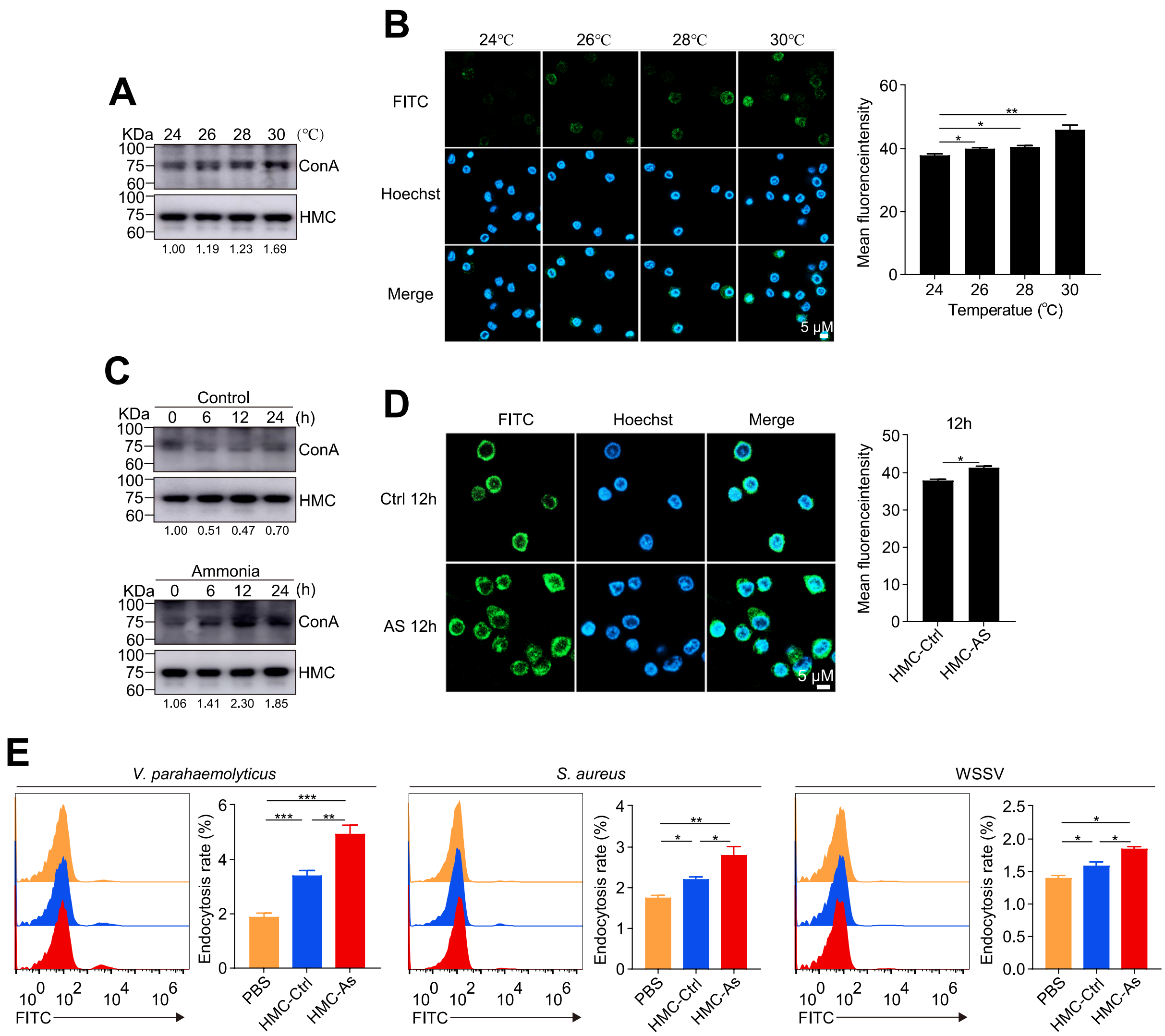

Fig. S5. Enhanced endocytosis of mannosylated plasma hemocyanin by hemocytes under high-temperature stress and ammonia stress. (A) Western blot analysis showing increased levels of mannosylated plasma hemocyanin (HMC) under different temperature stress conditions (24°C, 26°C, 28°C, and 30°C). (B) Microscopic images of FITC-labeled hemocyanin (FITC-HMC) endocytosed by hemocytes. Nuclei were stained with Hoechst 33342 (blue), and fluorescence intensity was quantified using ImageJ software. Scale bar = 5 μm. (C) Western blot analysis showing mannosylated plasma hemocyanin (HMC) levels under ammonia stress (100 mg/mL ammonia nitrogen) compared to the control group (0 mg/mL ammonia nitrogen). (D) Microscopic images of FITC-labeled HMC endocytosed by hemocytes. Scale bar = 5 μm. Nuclei were stained with Hoechst 33342 (blue). Mean fluorescence intensity was quantified using ImageJ software. (E) Relative endocytosis of FITC-labeled V. parahaemolyticus, *S. aureus*, and WSSV pre-incubated with control hemocyanin (HMC-Ctrl) or ammonia stress hemocyanin (HMC-AS), as determined by flow cytometry. Data are presented as mean ± SEM (n = 3). Representative immunoblot images are shown from at least two independent experiments. HMC, hemocyanin. Statistical significance was determined using Student’ s t-test (*$p < 0.05$, **$p < 0.01$, ***$p < 0.001$). Error bars represent S.E.
